# Nanoscale Mapping of Internal Magnetization Dynamics Reveals How Disorder Shapes Heat Generation in Magnetic Particle Hyperthermia

**DOI:** 10.1021/acs.nanolett.6c02907

**Published:** 2026-10-27

**Authors:** Elizabeth M. Jefremovas, Pauline Rooms, Álvaro Gallo-Córdova, María P. Morales, Frank Wiekhorst, Andreas Michels, Jonathan Leliaert

**Affiliations:** † Department of Physics and Materials Science, University of Luxembourg, 162A Avenue de la Faiencerie, L-1511 Luxembourg, Grand Duchy of Luxembourg; ‡ Institute for Advanced Studies, University of Luxembourg, Campus Belval, L-4365 Esch-sur-Alzette, Luxembourg; § Department of Chemistry, Ghent University, 9000 Ghent, Belgium; ∥ 69570 Instituto de Ciencia de Materiales de Madrid (ICMM-CSIC), Madrid 28049, Spain; ⊥ Physikalisch-Technische Bundesanstalt, D-10587 Berlin, Germany; # DyNaMat, Department of Solid State Sciences, Ghent University, 9000 Ghent, Belgium

**Keywords:** Magnetic hyperthermia, micromagnetic simulations, magnetic nanoparticles, heat release, intraparticle temperature difference

## Abstract

Magnetic particle hyperthermia relies on the efficient conversion of magnetic field energy into heat, yet the microscopic mechanisms governing this process within individual particles remain poorly understood. Here, we combine AC magnetometry experiments with dynamic micromagnetic simulations to connect microstructural features, magnetization dynamics, and macroscopic heat dissipation. We resolve heat generation at the intraparticle level, uncovering heterogeneous nanoscale heat-generation bursts with nanometer and nanosecond resolution. At a given particle size, grain size acts as an experimentally tunable parameter, balancing anisotropy disorder and pinning strength, thereby controlling both the magnitude and spatiotemporal distribution of heat release. Experiments show that flux-closure-dominated nanoflower architectures composed by larger grains generate more heat than their smaller-grain counterparts, whose deeper intraparticle pinning landscape redistributes heat generation over extended time windows. Together, our results provide a mechanistic framework linking nanoparticle microstructure to magnetic heating and establish design principles for optimizing nanoflowers as magnetic hyperthermia transducers.

## Graphical abstract

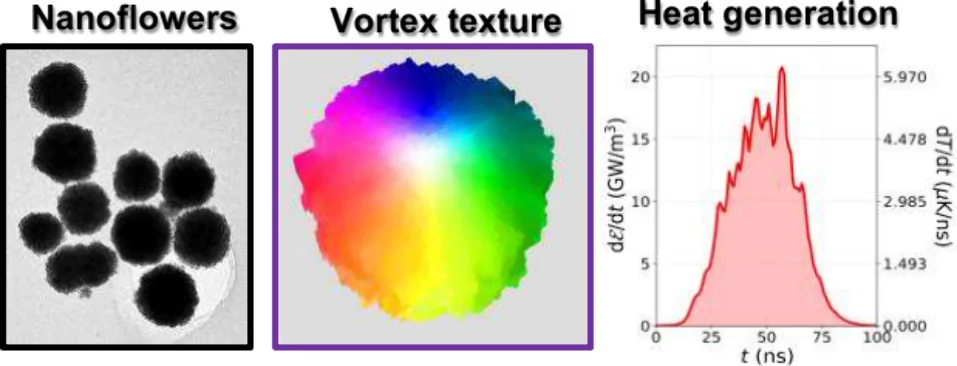

Iron oxide nanoparticles (IONPs) combine excellent magnetic properties, synthesis control, and biocompatibility, making them one of the most established magnetic nanomaterials for biomedical and technological applications.^1–4^ A particularly powerful feature of IONPs is their ability to operate as remote magnetic nanotransducers, converting energy delivered by external alternating (AC) magnetic fields into nanoscale functional responses. This capability underpins magnetic hyperthermia therapy (MHT), where magnetic losses are exploited to generate heat and disrupt cancer cell activity,^5,6^ and is increasingly relevant to broader field-activated strategies including magnetic particle imaging,^7^ neuromodulation,^8,9^ drug delivery,^10^ catalysis,^11^ and environmental remediation.^12^

Despite the extensive experimental demonstration of IONP heating performance,^5,13–16^ the microscopic origin of heat dissipation remains insufficiently resolved. A recent computational-experimental study has provided an important step forward by linking nonequilibrium nanoscale magnetization dynamics to macroscale calorimetric losses.^17^ However, this picture still relies on the macrospin approximation in which each nanoparticle is treated as being uniformly magnetized. Such an approach cannot capture the internal magnetic complexity of real nanoarchitectures, where defects, vacancies, crystallite boundaries, and polycrystalline disorder can generate inhomogeneous spin textures and strongly modify the macroscopic magnetic response.^18–22^ Consequently, the mechanistic link between nanoparticle microstructure, intraparticle magnetization dynamics, and magnetic heat generation remains largely elusive.

Here, we unravel this connection in defect-rich polycrystalline iron oxide nanoarchitectures by resolving how structural inhomogeneities determine the magnetic heat dissipation. We focus on maghemite nanoflowers (NFs), multicore nanoarchitectures with excellent hyperthermia performance,^13,14,23^ within a broader family of high-performing maghemite nanoarchitectures that also includes nanocube-based systems with clinically relevant heating efficiency.^24^ The structure of a NF, represented in Figure 1, comprises a multicore aggregate of mesocrystals, also referred to as *grains*, which act as individual crystallographic units. Both the NF diameter *d* and grain size *g*_*s*_ can be tuned via chemical synthesis,^12,16,25^ enabling control over the intraparticle degree of disorder. By combining experimental heating metrics and a micromagnetic framework allowing one to reproduce the morphological complexity and intraparticle crystallinity, we go beyond uniformly magnetized descriptions, capturing the magnetization dynamics of vortex-like textures under AC magnetic fields. This enables us to resolve the spatial and temporal landscape of intraparticle heat generation, identifying nanoscale heat-generation bursts associated with disorder-driven pinning and vortex-mediated reversal. The combination of the microscopic description with magnetic fluid hyperthermia experiments enables us to establish a direct link between structural disorder and internal magnetic states with macroscopic heating metrics. Together, our results reveal spin disorder as a functional engineering parameter governing magnetic heat release, providing a mechanistic basis for the rational optimization of magnetic induction heating applications.

**Figure 1:**
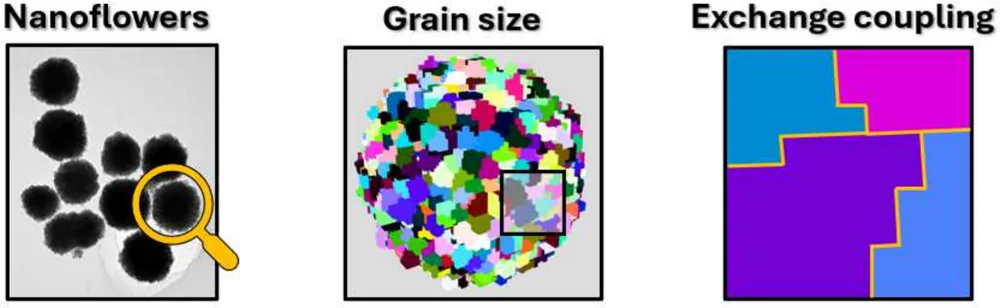
Iron oxide nanoflowers (NFs) are defect-rich polycrystalline nanoarchitectures formed by assembled mesocrystals, each behaving as a single crystallographic unit. Their intraparticle pinning landscape is modeled through tailorable grain size, randomly oriented grain anisotropy axes ${\mathrm{K⃗}}_{u}$ (represented in different colors), and reduced exchange coupling at intergrain boundaries (highlighted in yellow).

To link microstructural disorder to heat generation under magnetic hyperthermia conditions, we identify particle size, *d*, and grain size, *g*_*s*_, as the main physical control parameters. Together, they determine the number of constituent grains, *N*, and the balance between intergrain pinning and the anisotropy disorder associated with the grain anisotropy easy axes. Both *d* and *g*_*s*_ can be controlled experimentally through growth conditions.^23,25,26^ Importantly, *N* is a derived descriptor and does not uniquely determine the magnetic state: different combinations of *d* and *g*_*s*_ can yield similar *N* while supporting distinct magnetization textures and reversal dynamics. For the present NFs, increasing *d* drives the system from single-domain (*d* < 50 nm) toward a vortex regime with the latter displaying either a core-dominated (70 < *d* < 110 nm) or flux-closure-dominated reversal (*d* > 110 nm).^21^ In the former case, the vortex core is sufficiently large such that the minimization of its energy within an externally applied field determines the magnetization dynamics. In the latter case, found in larger particles, the flux-closure domains surrounding the vortex take over this role and dominate the magnetization dynamics.^21^ Because these different magnetic states exhibit intrinsically different reversal mechanisms, they also lead to different heat-generation dynamics.

Throughout the following discussion, the terms “larger-grain” and “smaller-grain” refer exclusively to the relative grain size *g*_*s*_ within NFs of fixed or comparable overall diameter *d*. For instance, *d* = 170 nm and *g*_*s*_ = 21 and 7 nm denote the larger- and smaller-grain realizations, respectively, while for *d* = 100 nm, the corresponding values are *g*_*s*_ = 15 and 4 nm.

Figure 2A–C displays the experimental heating metrics determined via the specific absorption rate, SAR, corresponding to four different NF ensembles. The NFs are considered in two comparative pairs: (*d*, *g*_*s*_) = (250, 25) nm and (*d*, *g*_*s*_) = (220, 10) nm have different nominal mean diameters but substantially overlapping size distributions, as determined by TEM, with *d* = 250 ± 20 nm and *d* = 220 ± 30 nm (see Supporting Information Table S1 and Figure S1), while (*d*, *g*_*s*_) = (170, 21) nm and (*d*, *g*_*s*_) = (170, 7) nm provide a direct comparison at the same mean NF diameter. The experimental results consistently indicate for the three different frequencies *f* a reduced heat generation for NFs with smaller grains (*g*_*s*_ = 10 and 7 nm) compared to the case of larger grains (*g*_*s*_ = 25 and 21 nm).

**Figure 2:**
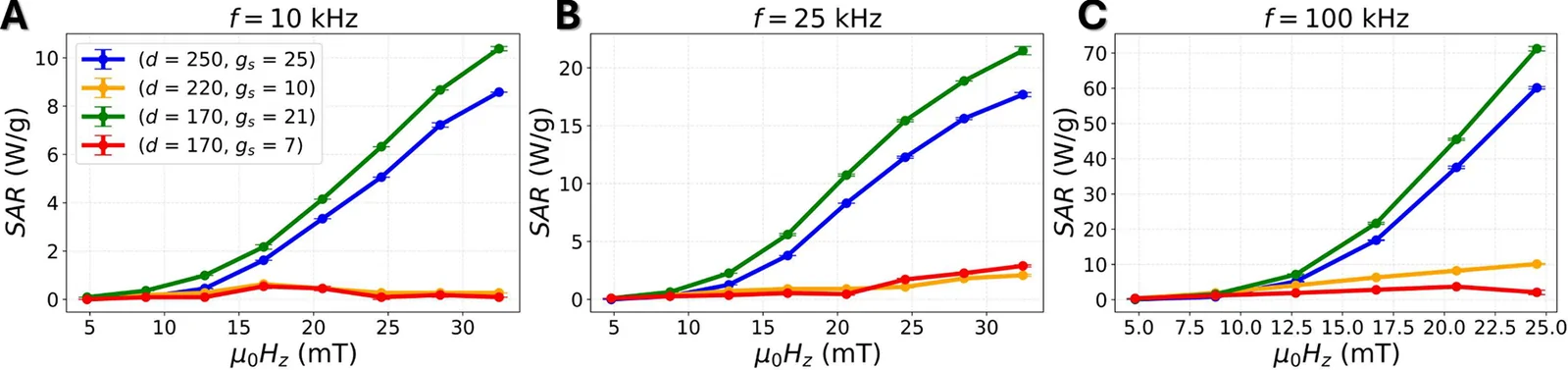
Specific absorption rate, SAR, vs field intensity, μ_0_ *H*_*z*_, for the 4 NF ensembles with diameter *d* = 250, 220, and 170 nm and grain size *g*_*s*_ = 25, 10, 21, and 7 nm measured at A) *f* = 10, B) *f* = 25, and C) *f* = 100 kHz. In all cases, the NFs with the smaller grain sizes *g*_*s*_ display almost negligible SAR throughout the explored field range.

The samples with larger grain size (250, 25) nm and (170, 21) nm develop a measurable SAR for μ_0_ *H*_*z*_ ≳ 12.7 mT, increasing its magnitude with μ_0_ *H*_*z*_ and *f*. For *f* = 10 and 25 kHz, the SAR­(μ_0_ *H*_*z*_) curves indicate a progressive approach to SAR saturation beyond μ_0_ *H*_*z*_ = 30 mT. At the highest experimentally accessed frequency, *f* = 100 kHz, the SAR of the larger-grain NFs continues to increase throughout the accessible field range, reaching approximately 60–70 W/g at μ_0_ *H*_*z*_ = 24.5 mT without evidence of saturation. This absence of clear saturation indicates substantial headroom for further improving performance at higher field amplitudes and/or frequencies (factor 2 under Hergt criteria^27^). This result is particularly encouraging for biomedical applications. For particle sizes *d* > 70 nm, the magnetization of the NFs is expected to adopt a vortex configuration.^21,28^ This is an advantageous configuration since it combines (i) a flux-closure pattern, which suppresses stray fields and can therefore mitigate dipolar interactions that promote particle aggregation and compromise *in vivo* heating performance,^29–33^ with (ii) a vortex core, which carries a net magnetization that can contribute to heating or serve as a vector for drug delivery.^13,23,32,34–37^ Our experimental observation that vortex-state NFs retain substantial heating efficiencies therefore identifies a favorable regime in which efficient magnetic heating is combined with a low-stray-field configuration, potentially favoring colloidal stability under physiological conditions.

Our experimental results reveal a key influence of the grain size on the heat dissipation with larger grains contributing more effectively compared to the smaller ones. We herein build further on the numerical side to capture the intraparticle mechanisms giving rise to such differences.

In order to unambiguously evaluate the influence of *g*_*s*_ on heat generation, we compare NFs with *d* = 170 nm, whose magnetization dynamics is dominated by flux-closure moments,^21^ with smaller NFs, *d* = 100 nm, where the dynamics is instead governed by the magnetic moments within the vortex core.^21^ This comparison allows us to extend the conclusions on grain-size effects to the whole vortex regime, beyond the specific flux-closure ensembles investigated experimentally in this work. For completeness, we have included in Supporting Information S5 and S6 the cases of *d* = 220 nm and *d* = 250 nm, which show qualitatively the same behavior as *d* = 170 nm.

Figure 3 includes *M*_*z*_ vs *H*_*z*_, together with the corresponding magnetic energy accumulated per AC cycle, $E_{acc}$ , corresponding to *d* = 170 nm with *g*_*s*_ = 7 and 21 nm and *d* = 100 nm with grain sizes *g*_*s*_ = 4 and 15 nm. These provide pairs of realizations with comparable numbers of constituent grains *N*. Specifically, the configurations (*d*, g*_s_*) = (100 nm, 4 nm) and (170 nm, 7 nm) comprise similar grain counts with *N* ≃ 8181 and *N* ≃ 7500, respectively, while the configurations (100 nm, 15 nm) and (170 nm, 21 nm) yield *N* ≃ 160 and *N* ≃ 278. The use of comparable grain numbers *N* across these size regimes enables one to extract and identify the different functional role of intraparticle disorder within the two magnetic dynamics regimes. The field direction was chosen as an arbitrary reference axis and does not represent a preferential structural direction of the NF. Accordingly, *M*_*z*_ denotes the magnetization component parallel to the applied field. Note that heat transport through the surrounding medium is not taken into account, and thus, our results correspond to an upper boundary for the heat release. The color-coded magnetization, realizations with smaller *g*_*s*_, showcase a sustained, progressive heat release over the whole reversal process (Figure 3A,E), whereas for the larger *g*_*s*_, rather than being uniformly distributed throughout the magnetization reversal, the heat release is localized in short bursts near the onset and completion of the magnetization reversal process (see Figure 3B,D), regardless of the vortex core- or flux-closure-dominated regimes. We identify the pinning of the vortex core by the grain-scale disorder as a key mechanism governing the magnetization reversal. During depinning and reconfiguration, the local magnetization becomes misaligned with the effective field **B**_eff_, giving rise to a dissipative torque whose associated energy-loss rate scales as $dE/dt\propto |m\times B_{e\mathrm{ff}}{|}^{2}$ . Consequently, the maximum heat generation occurs at those stages of the reversal where the misalignment between **m** and **B**_eff_ is largest. In particular, the multistep *M*–*H* trajectory observed in Figure 3B,D reflects successive reconfigurations of the internal magnetic texture with the stronger color-coded dissipation occurring during the sharper reversal events. Furthermore, smaller grain sizes *g*_*s*_ imply a larger number of constituent grains *N*, which generate a denser and more complex pinning landscape, characterized by a higher density of intergrain boundaries and a broader distribution of local anisotropy axes, which in turn, enhance the coherence and rigidity of the vortex core. As a consequence, for the vortex-core dominated NFs (*d* ≲ 110 nm^21^), the field-driven motion of a more rigid vortex core through this disordered landscape thus requires greater work against pinning forces, resulting in enhanced instantaneous energy dissipation, i.e., the instantaneous rate at which magnetic energy is converted into heat. On the other hand, the smaller amount of *N* of the larger grains result in a vortex core comparatively fragmented or strongly distorted, and the reversal proceeds through more local, incoherent rotations, which reduces the net torque and the associated energy dissipation. For the flux-closure-domain-dominated NFs, the situation inverts: smaller grain sizes result in a more homogeneous intraparticle pinning landscape with associated smaller energy barriers. As a result, the heat generation is reduced, as already observed in the experimental results shown in Figure 2.

**Figure 3:**
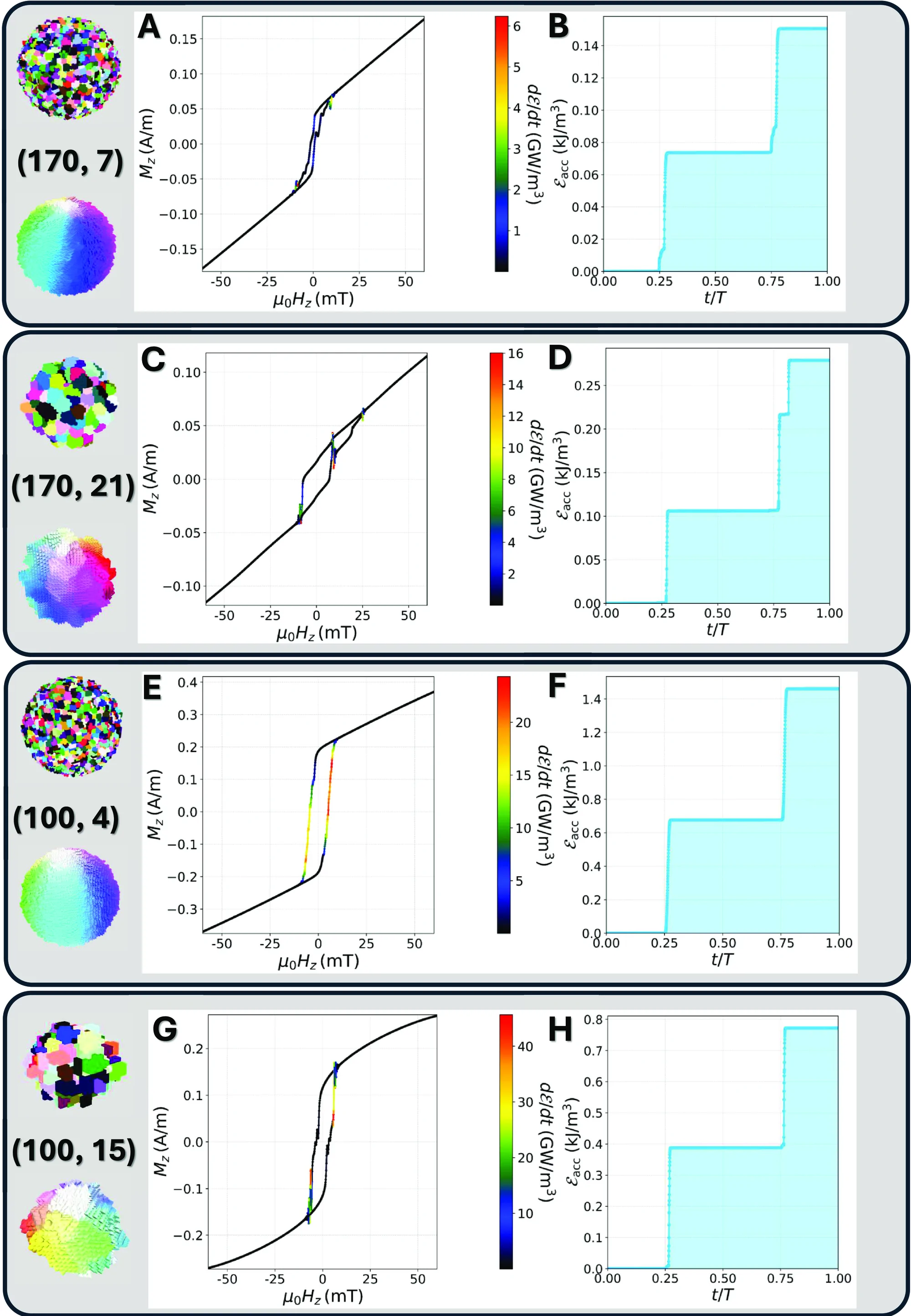
Magnetization reversal and energy released per cycle corresponding to fully coupled NFs (intergrain exchange parameter *k* = 1) with varying diameters and grain sizes: (A, B) *d* = 170 nm and *g*_*s*_ = 7 nm; (C, D) *d* = 170 and 21 nm; (E, F) *d* = 100 nm and *g*_*s*_ = 4 nm; and (G, H) *d* = 100 and 15 nm. (A), (C), (E), and (G): Magnetization as a function of applied field over one AC-field period τ = 1/300 ms. The magnetization traces are color-coded by the instantaneous heat generation with red highlighting the maximum. (B), (D), (F), and (H) Magnetic energy accumulated, $E_{acc}\left(t\right)$ , over τ = 1/300 ms. Left insets represent grain anisotropy distribution (top), where each color represents a ${\mathrm{K⃗}}_{u}$ direction, and the vortex configuration (bottom), where each color represents a direction of the magnetization.

The results consistently indicate that vortex core-dominated realizations deliver larger Δ*T* $\left(E_{acc}\right)$ compared to the flux-closure ones, regardless of the grain size, highlighting the dominant role of the reversal dynamics over the intraparticle grain boundaries. In this way, the fact that the magnetization reversal of flux-closure-dominated ensembles occurs partially perpendicular to the *z*-direction, whereas the vortex core-dominated ones follow a reversal path purely along the *z*-axis,^21^ smoothens the torque exerted by the driving field into the magnetization, resulting in a reduced heat generation for the larger flux-closure-dominated NFs. However, the intraparticle pinning landscape does affect the heat release. Within the vortex-regime, we detect a systematic increase with the intergrain exchange coupling parameter *k* for (*d*, *g_s_*) = (100 nm, 4 nm) (see Figure S4 in the Supporting Information for additional results with *k* = 0.25, 0.5, and 0.75). In this case, a larger intergrain exchange coupling yields a more robust and coherent vortex-core profile, which in turn results in an enhanced torque and related energy release. In contrast, within the flux-closure-dominated regime, the collective dipolar-mediated rotation smoothens the dynamics, weakening the dependence on *k* and, thus, on the grain size. We report however a larger heat generation (almost double) for the NFs with larger grains in agreement with the experimental results introduced in Figure 2. Indeed, thermal fluctuations, size polydispersity, and particle agglomeration prevent the achievement of a full quantitative equivalence between experiments and simulations. While our calculations consider heat dissipation from a single NF, experiments probe ensembles at Fe concentrations of ∼2–4 mg/cm^3^, corresponding to particle concentrations of $∼10^{11}NFs/cm^{3}$ . Ensemble effects, including size distributions^38,39^ and interparticle interactions arising from agglomeration,^29–31^ are therefore expected to influence the measured heating metrics. In addition, hydrodynamic degrees of freedom prevent a full quantitative equivalence between experiments and simulations. In particular, while the experiments are performed in magnetic fluid, the micromagnetic calculations consider fixed NFs and therefore exclude whole-particle Brownian rotation. For the present NF sizes, however, the characteristic Brownian relaxation times are substantially longer than the AC-field period, suggesting that Brownian rotation cannot follow the excitation on a cycle-by-cycle basis. The simulations therefore primarily resolve the intrinsic internal magnetization dynamics, while slower hydrodynamic effects may still contribute to the ensemble-averaged experimental response. Hence, despite the absence of quantitative agreement, our micromagnetic simulations performed at *f* = 300 kHz to keep the systematic parameter-space study computationally tractable reproduce the experimentally observed trends across the different nanoflower systems. This qualitative consistency validates the proposed physical picture and demonstrates that the dominant mechanisms governing heat generation are correctly identified. Incorporating thermal fluctuations, size polydispersity and interparticle interactions, together with experimentally matched excitation conditions, are natural following steps towards quantitative predictive capability.

Finally, our results provide the mechanistic understanding underlying the existence of nanoscale heat-generation bursts connected to MNPs, as evidenced in nanothermometry experiments.^40–42^ We estimated the corresponding temperature raise, Δ*T*, calculated from $E_{acc}$ as $\Delta T=\frac{E_{acc}}{\rho c_{p}}$ with *c*_*p*_ = 670 J/(kg·K).^43^ Our calculations indicate that Δ*T* reaches a maximum value of ≅0.436 mK for the most favorable case with *k* = 1 and (*d*, *g_s_*) = (100 nm, 4 nm) after one excitation period. Assuming that the heat accumulates per cycle, already after *t* = 0.1 s, the temperature increase at the NF shall reach Δ*T* ≅ 13 K, which is compatible with triggering cellular stress.^44^ Our estimations neglect heat diffusion from the NF into the surrounding medium and therefore represent an adiabatic upper bound rather than a prediction of the temperature reached after multiple excitation cycles. Future investigations explicitly coupling the present micromagnetic energy-dissipation framework to heat transport through the surrounding medium will be required to determine how heat generation at the single-particle scale translates into experimentally observable temperature rises.

After having examined the functional role of intraparticle magnetic disorder into macroscopic parameters, we resolve the heat generation microscopically, identifying the presence of nanoscale heat-generation bursts at intraparticle level, connecting them to the magnetization reversal stage at nanosecond time resolution.

Figure 4 showcases the spatial distribution of the local heat release at nanometer resolution at the instant of maximum dissipation for each configuration. Note that the bottom time Δ*t* represents the heat-generation window over which the heat is dissipated, which is different for each NF. The top axis includes the corresponding *t*/τ with τ = 3.33 μs for all cases. The maximum heat dissipation occurs when the vortex core is oriented perpendicular to the applied field along the *z* axis (see 3D magnetization plots in the middle panel of Figure 4), maximizing the instantaneous misalignment between the magnetization and the effective field **B**_eff_, which represents the total effective magnetic field acting on the magnetization, including in the present case exchange, demagnetization, anisotropy, and Zeeman energy contributions and, thus, the heat generation. We refer the reader to eq S2 included in the Supporting Information, where the explicit connection between heat generation and magnetization dynamics is shown. For the NFs with smaller grains and thus a larger number of constituents, the heat release is spatially distributed over multiple regions, reflecting a complex pinning landscape where the vortex core interacts with numerous grain boundaries and local anisotropy directions. This results in an extended dissipation pattern, consistent with a gradual and sustained reversal process. In contrast, NFs with larger grains exhibit highly localized and less dense nanoscale heat-generation bursts, indicating a rapid and collective reorientation of the magnetic texture. In this regime, the reduced pinning landscape allows the vortex state to collapse or rotate abruptly once the Zeeman term becomes the dominant factor within the effective field **B**_eff_.

**Figure 4:**
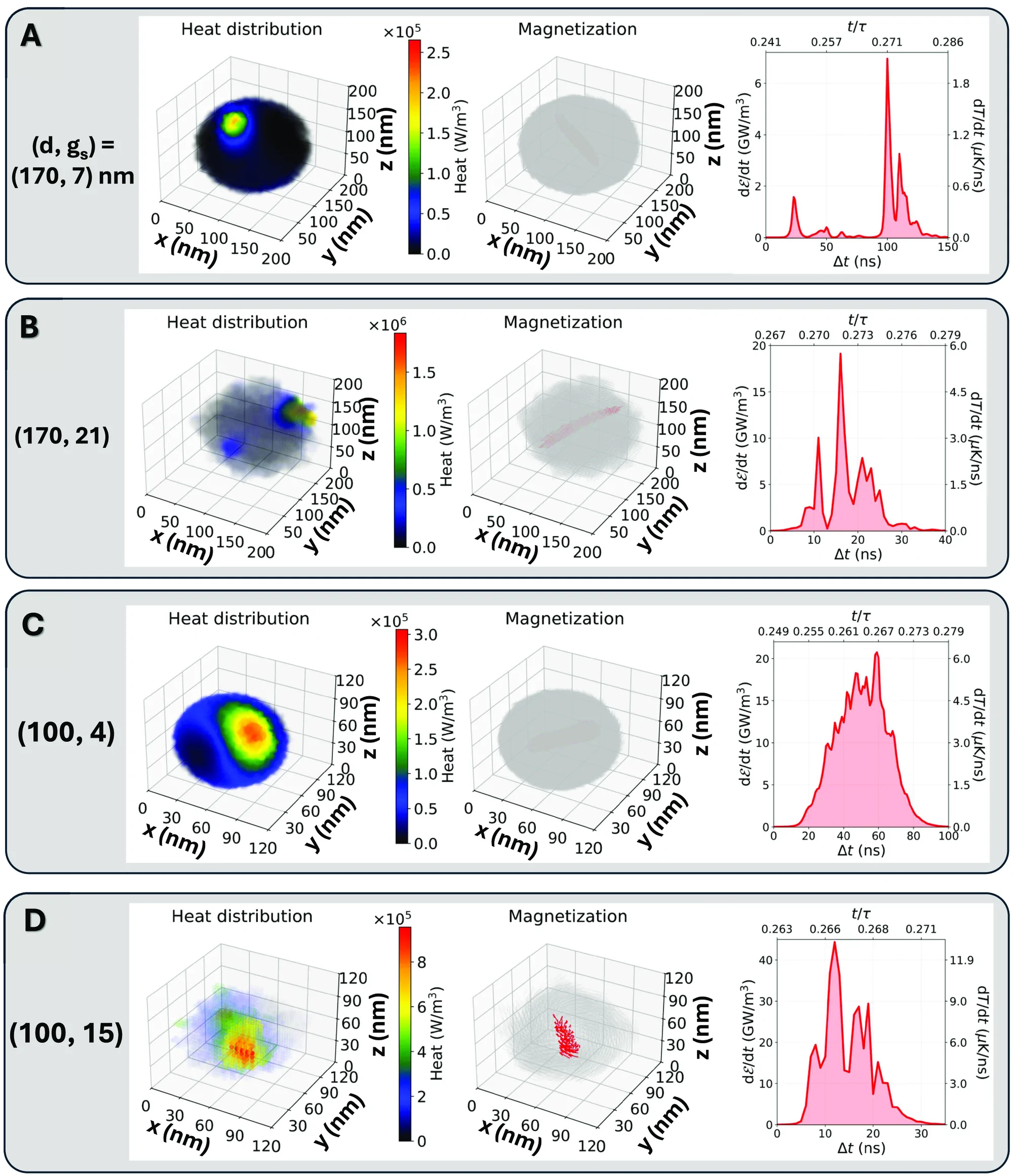
3D mappings of heat generation together with the corresponding 3D magnetization configurations and instantaneous heat release dE/dt and corresponding d*T*/d*t* as a function of time for (*d*, *g*_*s*_) = (170, 7), (170, 21), (100, 4), and (100, 15) nm ((A)–(D), respectively). Intergrain exchange coupling parameter is fixed to *k* = 1, and the external alternating magnetic field is applied at an amplitude of μ_0_ *h* = 62 mT and frequency *f* = 300 kHz. The color bar indicates the intensity of the heat release, ranging from black (minimum) to red (maximum). Magnetic moments within the vortex core are shown as red arrows, while flux-closure moments are represented in gray. The bottom time Δ*t* represents the heat-generation window over which the heat is dissipated, while the top axis includes the corresponding *t*/τ with τ = 3.33 μs.

Figure 4 also includes the instantaneous heat release dE/dt vs *t* together with its conversion to instantaneous temperature increase $\frac{dT}{dt}=\frac{1}{\rho c_{p}}\frac{dE}{dt}$ along with the visualizations of the heat and magnetization resolved at nanometer resolution (cell size) for *d* = 170 and 100 nm with *g*_*s*_ = 7, 21 and 4, 15 nm, respectively. As it can be observed, the heat release for the smaller grains is sustained over the whole reversal process, which we identified to happen at *t* ∼ 100 ns. The heat release happens for the larger grains; however, it was a factor 4 faster, *t* ∼ 25 ns, concentrated at the beginning of the reversal process. Such distinct temporal profiles of the heat release can be traced back to the reversal dynamics through eq S2 (see Supporting Information), which relates the instantaneous dissipation rate dE/dt to the torque exerted on the magnetization, as $dE/dt\propto {\left|m\times B_{e\mathrm{ff}}\right|}^{2}$ with **m** = **M**/*M*_*s*_ representing the normalized magnetization vector. In NFs with smaller grains, i.e., larger amount of intraparticle boundaries *N*, the dense and heterogeneous pinning landscape prevents rapid alignment of the magnetization with the effective field, sustaining moderate but nonzero values of ${\left(m\times B_{e\mathrm{ff}}\right)}^{2}$ over an extended time interval. As a consequence, the heat is released gradually throughout the reversal process, as indicated in the *M* vs μ_0_ *H*_*z*_ plots included in Figure 3. In contrast, NFs with larger grains offer a reduced pinning landscape, which reduces the rigidity of the vortex core. As a result, the strong initial misalignment of the vortex core with **B**_eff_ can be easily corrected, delivering a large value of the torque (**m** × **B**_eff_) over a short time interval. Following eq S2, this leads to an intense but shorter dissipation of energy, followed by a negligible contribution once the magnetization realigns with the effective field. It is also worth mentioning the striking differences that intraparticle magnetic disorder introduces to the heat generation distribution in both space and time domains. Figure S7 in Supporting Information includes the calculated heat generation of perfect *d* = 50 nm single-domain nanospheres. As observed, the heat release occurs at faster pace (within 5 ns) and spreads over the whole particle, which is contrast to the case of the NFs for which the grain anisotropy delays the heat release and the vortex magnetization concentrates the heat release at the vortex core.

The maximum value of the instantaneous heat release further reflects the interplay between reversal dynamics and internal microstructure. Among the systems studied, the largest instantaneous heat release happens for the (100 nm, 15 nm) and (170 nm, 21 nm) cases, while significantly lower maxima, reduced to less than 20%, are found for the smallest grains (100 nm, 4 nm) and (170 nm, 7 nm). This trend highlights that the instantaneous heat release benefits from the abrupt reorganization of the magnetic texture during reversal, yet such abrupt changes are not beneficial for the time-integrated energy release, as showcased in Figure 3. In fact, slower magnetization dynamics imposed by a richer pinning landscape lead to a sustained heat release distributed over time rather than to short-lived, intense peaks. Such a temporally extended dissipation not only is advantageous to deliver a larger generated energy per AC cycle, i.e., the total energy dissipated over one complete AC-field period, but also avoids highly localized power spikes and promotes a more homogeneous and controllable thermal response, which is particularly desirable for magnetic hyperthermia applications.^23^

Our heating maps provide direct visual evidence that the internal granular structure governs both the magnitude and temporal profile of the heat generation, directly linking microstructure to reversal dynamics and energy dissipation. On the spatial scale, we identify the pinning of the vortex core at intergrain boundaries as the primary source of localized heat release in clear contrast to the homogeneous particle case, where the entire particle’s magnetization contributes to heat generation. Although heat generation is spatially localized at the nanoscale, the resulting temperature inhomogeneity is expected to relax rapidly by thermal conduction within the particle. In the idealized adiabatic limit, the characteristic relaxation time of a thermal perturbation extending over a length scale *L* can be estimated as τ_th_ ∼ *L* ^2^/(π^2^α), with α = *k*/(ρ*c*_*p*_) being the thermal diffusivity. Using α ∼ 10^–6^m^2^s^–1^ and *L* ∼ 50 nm, τ_th_ ∼ 0.1 ns. Consequently, any intraparticle thermal gradients associated with the spatially localized energy dissipation are expected to be extremely short-lived.

On the temporal time-scale, our results uncover that the heat generation is not continuous in time but instead occurs in intense transient bursts associated with magnetization reversal events. In both nanoflowers and homogeneous particles, the heat is generated for *t* ∼ 100 ns during each AC excitation cycle of ∼3.3 μs. This pulsed dissipation regime fundamentally differentiates magnetic nanoparticles from plasmonic nanoheaters, where heat generation is sustained continuously during illumination.^45^

Overall, these findings establish a direct connection between structural disorder, intraparticle magnetization, vortex dynamics, and transient heat generation, providing a physically grounded framework for understanding how micromagnetic textures control both the spatial and temporal characteristics of magnetic heat generation at the nanoscale.

In this work, we link microstructural features to macroscopic heating performance by resolving the spatiotemporal magnetization dynamics of iron oxide NFs, thereby connecting intraparticle magnetic defects to heat generation at the particle scale. Starting from the experimental observation that NFs with larger grains deliver better heat release, we use a micromagnetic framework to capture the influence of intraparticle magnetic disorder into the macroscopic heat release and to localize spatially and temporally the occurrence of nanoscale heat-generation bursts at intraparticle level.

By using the grain size as an experimentally controllable parameter, we have determined the crucial influence of structural disorder into the heat generation. Whereas the smaller grains offer a larger collection of intergrain pinning boundaries, which in principle should on its own deliver a larger energy release, they smoothen at the same time the anisotropy energy landscape, which facilitates core mobility through hopping between local anisotropy directions, highlighting the critical balance between both pinning and anisotropy landscapes. As a result, the smaller grains impose (i) a larger frequency and field amplitudes to trigger a heat release, as observed in the experimental results, and (ii) a heat release that balances the benefits of deeper pinning landscapes with the detrimental effect of weaker anisotropy landscape.

We have shown how NFs with larger grains within the flux-closure dominated regime deliver heat comparable to smaller commercial counterparts^13^ with the key advantage of being less prompt to agglomeration. Samples with smaller grains, despite offering a larger pinning potential in terms of intergrain boundaries, lose their heating power by offering a smoother anisotropy landscape, highlighting the dominant role of the latter over the intergrain pinning in the heating response. Furthermore, our experiments allowed us to underscore the role of thermal fluctuations, which do not change the qualitative trends, yet they effectively reduce the energy demands on the magnetic field excitation, showing larger heat release at smaller frequency and field amplitudes compared to our micromagnetic athermal simulations.

The larger heating performance of vortex core-dominated NFs compared to the flux-closure-dominated counterparts support the *d* = 100–120 nm size windows for the hyperthermia “sweet spot”.^21^ Within the flux-closure-dominated regime, both our experiments and simulations reveal a larger energy dissipated for realizations with larger grains at the experimentally measured fields, providing a guide for the rational design of optimized nanotransducers. The results further highlight larger grains yielding a more localized distribution of “heat generation spots”, along with heat generation happening at a faster pace, whereas smaller grains spread dissipation over broader regions of the particle and over longer time windows. This spatiotemporal resolution provides a practical design handle: it resolves at intraparticle level where heat is generated and for how long the release is sustained, thereby enabling informed choices of microstructure depending on whether rapid, concentrated heating or more distributed, sustained dissipation is desired. Future work should bridge the micro–macro gap in magnetic particle hyperthermia by explicitly incorporating heat diffusion across relevant length and time scales, which is expected to critically influence the experimental observation of localized nanoscale heat-generation bursts at the ensemble level. In this way, while nanothermometry experiments based on fluorescent probes indicate a significant temperature rise at the immediate vicinity of the MNPs,^40–42^ macroscale X-ray thermometry reports negligible particle–medium temperature differences.^46,47^ We hypothesize that this apparent discrepancy arises actually from the fundamentally different spatiotemporal resolutions of these techniques, namely, local particle-level measurements versus ensemble-averaged observations, which effectively smooth out transient and spatially confined thermal gradients.

We can anticipate that, for MHT applications, slower magnetization dynamics imposed by a rich pinning landscape leading to a sustained heat release distributed over time is preferred over short-lived, intense peaks. Indeed, a natural next step is to develop a multiphysics framework coupling the present micromagnetic energy-dissipation predictions to simulations of heat transport in the surrounding medium, enabling a quantitative connection between nanoscale heat-generation bursts and experimentally observable temperature increases.

## Supplementary Material



## Notes

Detailed explanations of the material and methods, structural characterization of the experimentally measured nanoflowers (transmission electron microscopy and X-ray diffraction); extended discussions on the role of thermal fluctuations in the simulations; experimental AC hysteresis loops from which the SAR is extracted; magnetization vs applied field and Δ*T* vs *t* for different intergrain coupling parameters, *k*; temperature mappings and heat release for (*d*, *g*_*s*_) = (220, 10) and (250, 25) nm; dissipated energy vs time over a whole AC cycle; temperature mappings, heat release, and magnetization reversal of a representative single-domain spherical nanoparticle (PDF)
