# Supplementary material for: Nanoscale Mapping of Internal Magnetization Dynamics Reveals How Disorder Shapes Heat Generation in Magnetic Particle Hyperthermia
Source: Nano Lett. 2026 Oct 27;26(44):15240–9. doi: 10.1021/acs.nanolett.6c02907 (PMC13761701; doi:10.1021/acs.nanolett.6c02907)
Supplement: Supplementary file 1 [file nl6c02907_si_001.pdf]

Supporting Information to: Nanoscale mapping of internal magnetization dynamics reveals how disorder shapes heat generation in magnetic particle hyperthermia

Elizabeth M. Jefremovas*[1,2], Pauline Rooms[3], Álvaro Gallo-Córdova[4], María P. Morales[4], Frank Wiekhorst[5], Andreas Michels[1], and Jonathan Leliaert[6]

[1]Department of Physics and Materials Science, University of Luxembourg, 162A Avenue de la Faiencerie, L-1511 Luxembourg, Grand Duchy of Luxembourg
[2]Institute for Advanced Studies, University of Luxembourg, Campus Belval, L-4365 Esch-sur-Alzette, Luxembourg
[3]Department of Chemistry, Ghent University, 9000 Ghent, Belgium
[4]Instituto de Ciencia de Materiales de Madrid (ICMM-CSIC), Madrid 28049, Spain
[5]Physikalisch-Technische Bundesanstalt, D-10587 Berlin, Germany
[6]DyNaMat, Department of Solid State Sciences, Ghent University, 9000 Ghent, Belgium

*Email: elizabeth.jefremovas@uni.lu

Abstract

This Supporting Information includes detailed explanations on the material and methods, the structural characterization of the experimentally measured nanoflowers (transmission electron microscopy and X-ray diffraction); extended discussions on the role of thermal fluctuations in the simulations; experimental AC hysteresis loops, from which the SAR is extracted; magnetization vs. applied field and ΔT vs. t for different intergrain coupling parameter k; temperature mappings and heat release for $(d, g_s) = (220, 10)$ and $(250, 25)$ nm; dissipated energy vs. time for over a whole AC cycle; and temperature mappings, heat release and magnetization reversal of a representative single-domain spherical nanoparticle.

Materials and methods

Experimental methods

Maghemite nanoflowers with the smaller grains (7 nm and 10 nm) were synthesized following a scaled-up version of our previously reported modified solvothermal polyol protocol [1]. Briefly, 4.37 g of iron(III) chloride hexahydrate ($FeCl_3{\cdot}6H_2O$, ≥98%, Sigma–Aldrich) were dissolved in 870 mL of ethylene glycol under magnetic stirring at 100 °C for 1 h. Subsequently, 28.4 g of sodium acetate and poly(vinylpyrrolidone) (PVP, $M_\mathrm{w} \approx 40\,000$, Sigma–Aldrich) were added to the solution. The amount of PVP was varied to tune the particle size: 90 g were used to obtain nanoflowers with an average diameter of ≈ 170 nm, whereas 45 g were used to produce larger particles ($d \approx 220$ nm). The mixture was stirred for ≈ 30 min until complete dissolution of the reagents, yielding a homogeneous precursor solution. The solution was then transferred to a Teflon-lined stainless-steel autoclave (1000 mL capacity) and heated at 200 °C for 16 h. After naturally cooling to room temperature, the resulting particles were magnetically separated and washed several times with ethanol.

The obtained particles were subsequently subjected to an acidic oxidation treatment. First, the particles were washed with 300 mL of nitric acid (HNO_3, 65%; Sigma–Aldrich) solution (2 M) and magnetically separated. They were then redispersed in 200 mL of iron(III) nitrate nonahydrate ($Fe(NO_3)_3{\cdot}9H_2O$,

$\geq$99.95%; Sigma–Aldrich) solution (0.6 M) and heated at 90 °C for 1 h. After cooling to room temperature, the particles were magnetically separated again, washed with 300 mL of HNO_3 (2 M), rinsed three times with an acetone/water mixture (30:70 v/v), and finally dried at 50 °C overnight.

Nanoflowers with larger grains (21nm and 25 nm) were synthesized under analogous solvothermal conditions. For particles with an average diameter of $d = 170$ nm, 10.4 g of $FeCl_3 \cdot 6H_2O$ and 3.2 g of trisodium citrate dihydrate (99%; Sigma Life Siences) were dissolved in 320 mL of ethylene glycol ($\geq$99%, Sigma–Aldrich), followed by the addition of 20 g of sodium acetate trihydrate ($\geq$99%, Sigma–Aldrich) under magnetic stirring. For larger particles ($d \approx 250$ nm), 21.62 g of $FeCl_3 \cdot 6H_2O$ and 6 g of trisodium citrate were dissolved in 376 mL of ethylene glycol, after which 40 g of sodium acetate trihydrate were added. In both cases, the mixtures were vigorously stirred for 30 min, transferred into a Teflon-lined aluminium autoclave (1000 mL), and heated at 200 °C for 16 h. After natural cooling to room temperature, the resulting black precipitate was magnetically separated and washed three times with distilled water by magnetic decantation. The final product was subsequently subjected to the acidic oxidation treatment described above and dried at 50 °C overnight.

Transmission electron microscopy (TEM) was performed to determine the morphology and particle-size distribution of the NFs. X-ray diffraction (XRD) was performed to identify the phase and nanocrystallite (grain) size of the NFs. Further details are included in Supporting Information S1.

AC magnetometry was performed using a commercial AC magnetometer system ("AC Hyster", Nanotech Solutions, Spain). The system is designed for characterising liquid nanoparticle samples of 40 μL. Hysteresis loops have been measured for the 4 different NF samples of several diameter and grain sizes: $d = 170$ nm, with grain sizes of $g_s = 21$ and 7 nm, and $d = 220$ and 250 nm, with $g_s = 10$ and 25 nm, respectively, at three different frequencies, $f = 10$, 25, and 100 kHz, with AC field amplitudes from $\mu_0 h_{\mathrm{AC}} = 3.83$ kA/m to 25.81 kA/m *i.e.*, all within the clinical safety limits $h \cdot f \leq 5$ GA/ms [2, 3].

Micromagnetic description of maghemite nanoflowers

Micromagnetic simulations were performed using Mumax3[4]. The NFs were generated starting from a cubic simulation region to which a Voronoi tessellation was applied to divide the volume into discrete regions corresponding to individual material grains [5]. A perfectly spherical geometry with an initial diameter d was then overlaid on this tessellated cube. Only the grains whose Voronoi centers were located inside the sphere were retained as part of the particle geometry, while grains with centers outside the sphere were removed. This procedure yields a nanoflower-shaped particle composed of multiple irregular grains. Intra-particle disorder was included by assigning a random uniaxial anisotropy direction K_u to each grain and by modulating the inter-grain exchange coupling at grain boundaries by a factor k. Additional details on the simulations are included in the Supporting Information and Fig. S4 therein. As shown in that S4, there is excellent agreement between hysteresis loops obtained with and without thermal fluctuations.
Critically, we identify both parameters to be controlled by an experimentally-tailorable parameter, the grain size [1, 6], which effectively determines (i) the intra-particle regions sharing the same K_u; and (ii) the amount of intra-particle grain boundaries, which determines the pinning landscape. The intra-particle pinning was captured by modelling the magnetic coupling between adjacent grains via rescaling the exchange parameter A at the grain boundaries by a factor k lying between 0 and 1. Material parameters typical for iron oxide were used, including saturation magnetization $M_s = 400$ kA/m [7, 8], exchange stiffness $A = 10$ pJ/m [9], uniaxial magnetocrystalline anisotropy $K_u = 10$ kJ/m^3 [7, 10–12] and Gilbert damping parameter $\alpha = 0.1$ [13, 14]. A time-dependent magnetic field $h(t) = h_{\mathrm{AC}} \cos(2\pi f\, t)$ is applied along the z-axis direction, with $f = 300$ kHz, to calculate the heat losses per cycle. The field direction was chosen as an arbitrary reference axis and does not represent a preferential structural direction of the NF. Accordingly, M_z denotes the magnetization component parallel to the applied field. Using Mumax3, we numerically integrate the Landau-Lifshitz-Gilbert (LLG) equation:

$$\frac{d\mathbf{m}}{dt} = -\frac{\gamma}{1+\alpha^2}\left[\mathbf{m} \times \mathbf{B}_{\mathrm{eff}} + \alpha\, \mathbf{m} \times (\mathbf{m} \times \mathbf{B}_{\mathrm{eff}})\right], \tag{S1}$$

and link the torque exerted on the magnetization to the magnetic energy dissipated via Eq. S2 [15]:

$$\frac{d\mathcal{E}}{dt} = \frac{\alpha\gamma M_s}{1+\alpha^2}\left(\mathbf{m} \times \mathbf{B}_{\mathrm{eff}}\right)^2 \tag{S2}$$

Here, $\mathbf{m} = M/M_s$ represents the normalized magnetization vector and $\mathbf{B_{eff}}$ is the total effective magnetic field acting on the magnetization, including in the present case exchange, demagnetization, anisotropy and Zeeman energy contributions. The term $\mathbf{m} \times \mathbf{B_{eff}}$ therefore describes the torque driving the magnetization dynamics.

To date, this expression has only been applied to nanoparticles within the macrospin approximation [16, 17]. Our work moves a step forward by applying it to the individual finite-difference cells in the simulation to investigate the heat dissipation in nanoparticles with internal magnetization inhomogeneities, for which large-scale simulations are performed. This dissipated energy can be transformed into the specific loss power or, equivalently, specific absorption rate, SAR[1] parameter quantifying the heating performance from the hysteresis losses, via:

$$SLP = \frac{f}{\rho}\mu_0 \oint M(H)\,\mathrm{d}H, \tag{S3}$$

and thus:

$$SLP = \frac{f}{\rho}\mu_0 \oint M(H)\,\mathrm{d}H = \frac{f}{\rho}\mathcal{E}^{(V)}_{\text{cycle}} \tag{S4}$$

Being ρ the density of the material, which for the present case of maghemite, is $\rho = 4.9$ g/cm^3 [18].

Structural characterization

Transmission electron microscopy (TEM) was performed using a JEOL JEM 1010 electron microscope at an accelerating voltage of 100 kV. For sample preparation, a diluted suspension of NFs was deposited onto an amorphous 300 micron meshed carbon-coated copper grid, followed by evaporation at room temperature. The particle-size distribution was analyzed using the standard software ImageJ [19] for digital electron microscope image processing, assimilating the particle diameter as the largest internal dimension. Data for an approximate number of at least 200 particles were acquired and fitted to a lognormal distribution, allowing for the calculation of the mean particle size and characterization of the particle size distribution. Figure S1 includes representative TEM images together with the corresponding statistical results for mean particle size, included also in Table S .

To identify the phase and crystalline structure of the NFs, X-ray diffraction (XRD) measurements were performed using a Bruker D8 Advance diffractometer equipped with a graphite monochromator and a Cu-Kα source ($\lambda = 1.5406$ Å). The XRD patterns were recorded at room temperature and ambient pressure in the 2θ range from 20° to 70°. The results, that can be inspected in Figure S1, where the most intense peaks have been indexed according to their Miller indices, confirm a single spinel iron-oxide phase, consistent with γ-Fe_2O_3/Fe_3O_4. Considering the post-synthetic oxidative treatment, the phase is assigned to maghemite. To estimate the mean crystal size, corresponding to the size of the grains, Scherrer's equation was used [20],

$$D = \frac{K\,\lambda}{\beta\cos\theta} \tag{S5}$$

where K is typically taken as 0.9 for nanoparticles and β represents the full width at half-maximum of the most intense peak, which in our case corresponds to the (3 1 1) reflection. The results for grain size are also included in Table S .

Magnetic Fluid Hyperthermia experimental measurements

In order to determine experimentally the heating efficiency of the nanoflowers, we have performed AC hysteresis loops measurements and calculated their Specific Absorption Rate (SAR) from the hysteresis losses associated. The AC hysteresis loops have been measured for 4 different NF samples of several diameter and grain sizes: $d = 170$ nm, with grain sizes of $g_s = 21$ and 7 nm, and $d = 220$ and 250 nm,

[1] Although a distinction between SLP (intrinsic heat generation) and SAR (experimental quantification of heat generation) is sometimes distinguished on conceptual grounds, both metrics are conceptually equivalent and quantify the same underlying heating process. Their formulation differs only in their normalization factor, with SAR defined per mass concentration in the medium (c) and SLP per intrinsic material density (ρ).

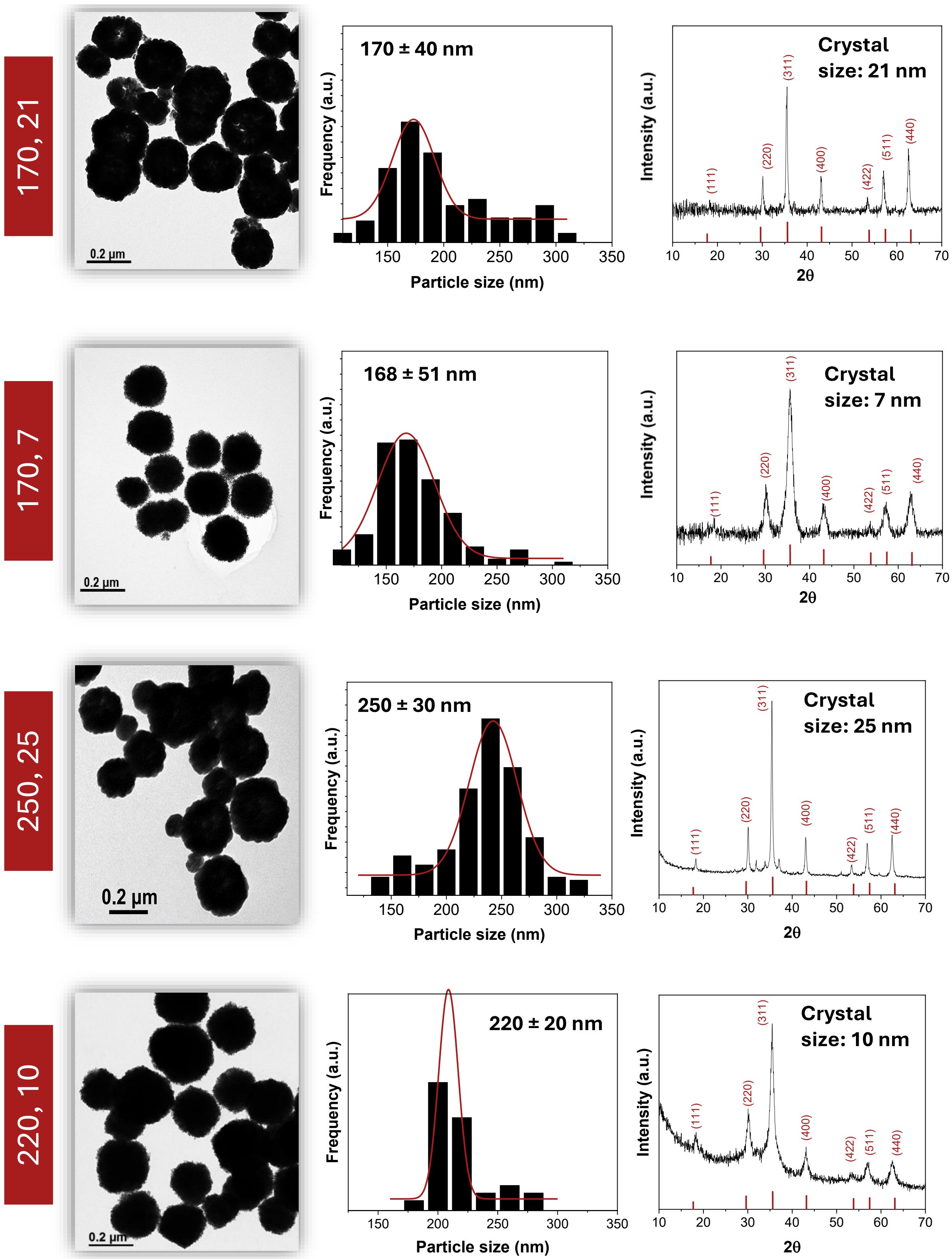

Figure S1: Representative TEM images, for the four different batches of NFs, along with their size distribution analysis, fitted to a log-normal distribution, and XRD patterns.

Table S1: Structural parameters of the studied samples: Mean diameter $\langle d \rangle$ and corresponding standard deviation σ, and grain size g_s.

Sample	$\langle d \rangle$ (nm)	σ (nm)	g_s (nm)
(170, 21)	170	40	21
(170, 7)	168	51	7
(250, 25)	250	20	25
(220, 10)	220	30	10

with $g_s = 10$ and 25 nm, respectively. The AC loops have been measured at three different frequencies, $f = 10$, 25, and 100 kHz, with AC field amplitudes from $h_{AC} = 3.83$ kA/m to 25.81 kA/m *i.e.*, all within the clinical safety limits $h \cdot f \leq 5$ GA/ms [2, 3]. These loops are included in Fig. S2, where it can be seen how the AC loops become wider at increasing the field amplitude h_{AC}, evolving from the narrow and elongated lancet shape at low fields, with low hysteresis losses and associated heating efficiencies, to wider and square-like loops of increasing heat losses.

A more insightful comparison emerges when contrasting samples with smaller grain sizes ($g_s = 7$ and 10 nm) to those with larger grains ($g_s = 21$ and 25 nm). While the former exhibit no measurable coercivity under any tested field amplitude or frequency, the latter progressively develop broader and more square-shaped hysteresis loops, reaching $\mu_0 H_c \cong 6.3$ mT at applied fields of 16.39 kA/m for $f = 10.0$ kHz and 19.53 kA/m for $f = 100.0$ kHz. This behaviour is illustrated in Fig. S2, where we show the loops acquired at $h_{\mathrm{AC}} = 19.53$ kA/m for all four samples across several frequencies.

As seen in the figure, the samples with $g_s = 21$ and 25 nm display nearly identical hysteresis loop shapes: wide, square loops with $\mu_0 H_c \cong 6.3$ mT and maximum magnetization values of $M \cong 40\ \mathrm{A\,m^2\,kg^{-1}}$, exhibiting only minor frequency dependence. In contrast, samples with $g_s = 7$ and 10 nm generate loops with almost negligible coercivity. Their maximum magnetization also differs: for particles of $d = 220$ nm, the saturation magnetization is approximately 1.2 times higher than in the $d = 170$ nm nanoflowers.

At the highest measured frequency, $f = 100$ kHz, the small-grain samples display only a marginal coercivity of $\mu_0 H_c \cong 1$ mT and 0.2 mT, respectively. This indicates that such small grains do not efficiently activate hysteresis losses at sub-hundred-kilohertz frequencies.

The role of thermal fluctuations in the simulations

Our micromagnetic simulations under frequency-dependent AC magnetic fields were performed without explicitly including a stochastic thermal field associated with finite-temperature fluctuations. This choice is motivated by computational feasibility and by the physical regime addressed in this work. Incorporating a thermal noise term within stochastic Landau–Lifshitz–Gilbert dynamics would dramatically increase the computational cost and prohibit the large-scale parameter study presented here, while providing limited additional insight into the dominant magnetization-reversal mechanisms and energy dissipation processes of interest.

The nanoflowers considered in this study are well above both the superparamagnetic and single-domain limits. The superparamagnetic diameter can be estimated as

$$d_{\mathrm{SPM}} \approx \left(\frac{6 \cdot 25 \cdot k_B T}{\pi K_u} \right)^{1/3} \approx 27 \text{ nm},$$

assuming $\tau = \tau_0 \exp(K_u V / k_B T)$ with $\ln(\tau/\tau_0) = 25$ at room temperature [21]. The single-domain limit can be approximated as $d_{\mathrm{SD}} \approx 7.2\, l_{\mathrm{ex}}$ [22], corresponding to ~ 51 nm for maghemite nanoflowers [23]. Within the investigated size range and excitation frequencies (10–300 kHz), the magnetization dynamics is therefore neither governed by Néel nor by Brownian relaxation. Instead, the response evolves from thermally assisted diffusion at low fields to field-induced crossing of energy barriers at larger amplitudes, resulting in a hysteretic regime [24–27], where heat release is primarily driven by the applied field with minor influence of thermal fluctuations on the absolute figures.

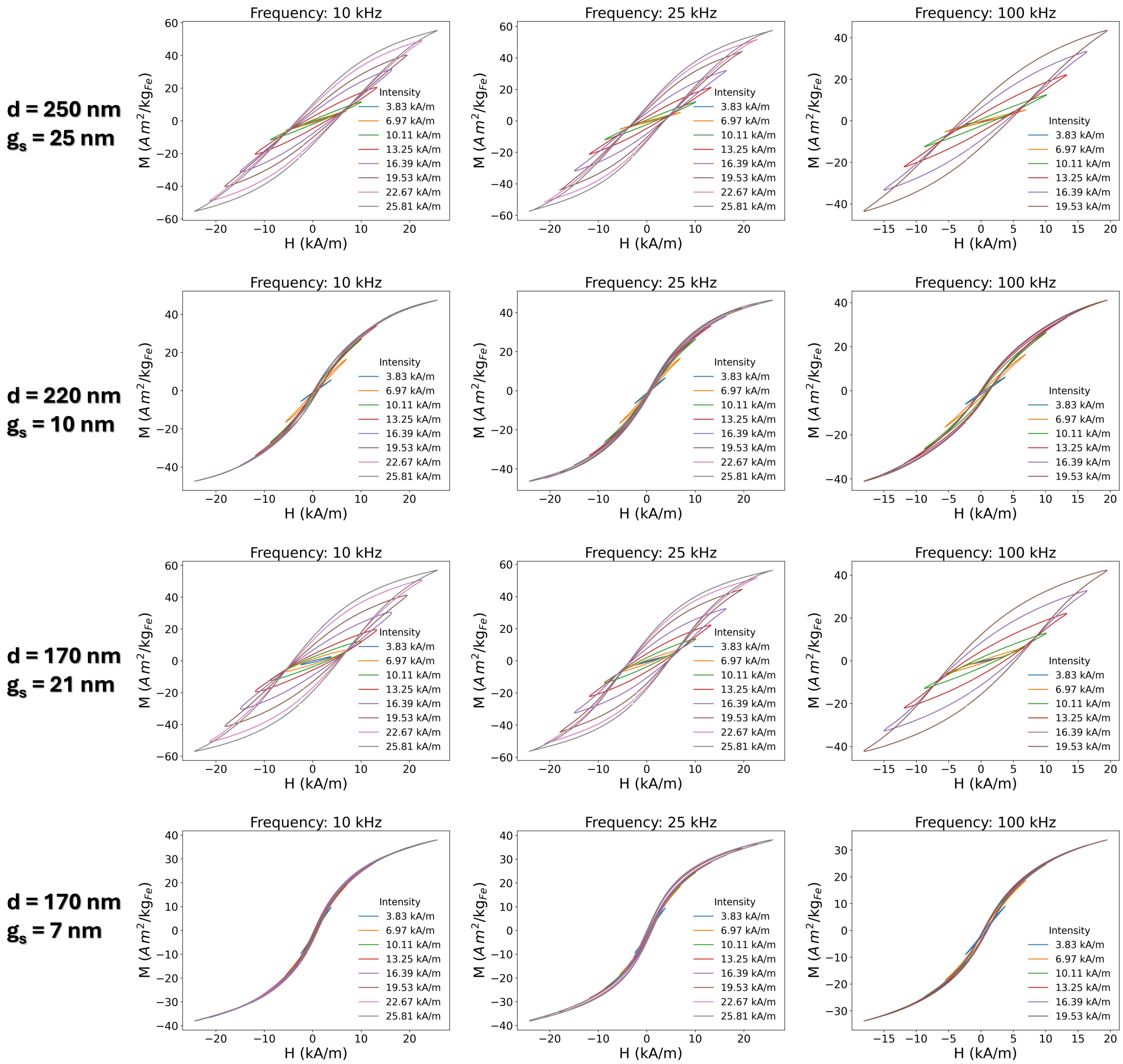

Figure S2: AC hysteresis loops measured for the NFs with d = 250, 220 and 170 nm with grain sizes of $g_s = 25, 10, 21$ and 7 nm (top to bottom), at f = 10, 25 and 100 kHz, with AC field amplitudes up to h_{AC} = 25.81 kA/m, respectively.

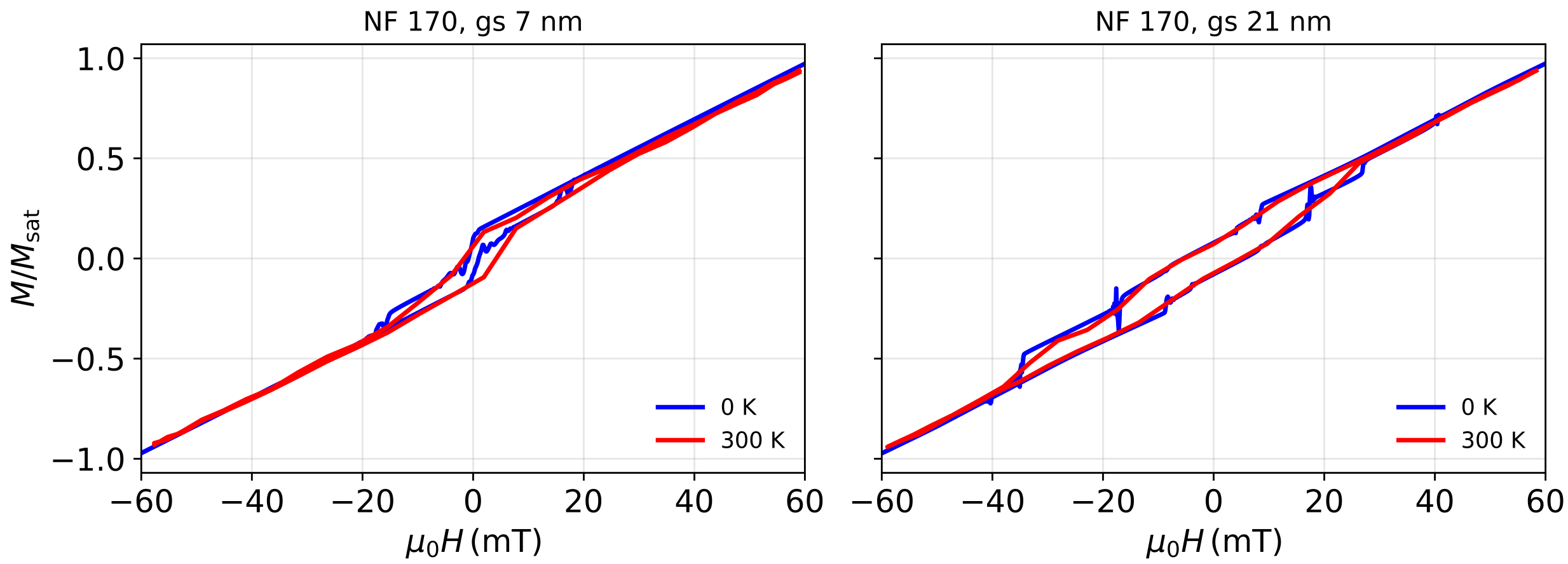

Figure S3: Hysteresis loops calculated for $d = 170$ nm NFs with $g_s = 7$ and 21 nm, respectively, at $T = 0$ K and 300 K. Driving field parameters are $f = 300$ kHz and $h_{AC} = \pm 49.33$ kA/m. No significant changes in the coercivity are observed.

To assess the impact of finite temperature on the extracted energy losses, we directly compared hysteresis loops obtained from simulations performed at $T = 0$ K and $T = 300$ K, with AC field excitation of $f = 300$ kHz and maximum field amplitude of $h_{AC} = \pm 49.33$ kA/m. Figure S3 presents averaged loops (10 realizations) for NFs of $d = 170$ nm, combined with grain sizes of $g_s = 7$ nm and 21 nm. In all cases, the loops display a similar qualitative behaviour: in the loops at $T = 0$ K, the magnetization exhibits some jumps, revealing the pinning of the vortex core at the inter-grain boundaries while the magnetization reversal takes place. Those jumps are suppressed at $T = 300$ K, where the thermal fluctuations provide an extra energy to overcome the pinning energy barriers, resulting in a smoother magnetization reversal with no pinning. However, the loop coercivity remains almost constant at both temperature values, constraining the effect of thermal fluctuations to the magnetization dynamics, yet with little to negligible effect on the macroscopic magnetic energy stored per cycle. This demonstrates that the dominant dissipation mechanism is field-driven, with minimal influence of thermal effects under the tested conditions.

Influence of intergrain exchange on the magnetization and ΔT

Figure S4 evaluates the influence of the intergrain exchange parameter k into the instantaneous $d\mathcal{E}/dT$ and ΔT values. As expected, the flux-closure-dominated NFs showcase a negligible influence of k into both $d\mathcal{E}/dT$ and ΔT, given that the magnetization dynamics is dominated by the stray-field term, with little influence of the vortex core [23]. For the vortex core-dominated nanoflowers, we observe that increasing the coupling k in between neighbouring grains extends the generation of the heat over a larger field range, consequence of the increased rigidity of the vortex core, and delivering larger ΔT ($\mathcal{E}_{\mathrm{acc}}$). This effect becomes particularly noticeable in the case of the smaller grains, which contain a larger amount of intra-particle boundaries (see Figure S4(**C**)).

Simulations for larger NFs: $d = 220$ nm and 250 nm

Figures S5 and S6 depict the results of $(d, gs) = (220, 10)$ and $(250, 25)$ NFs. Akin to the simulations in the main text, the energy accumulated per cycle is a factor 6 larger for the larger grains compared to the smaller counterparts, in agreement with the simulations and experiments. The heat release for NFs with smaller grains is aswell spatially-located around the vortex core and extends over a longer period of time ($t \sim 100$ ns) compared to the realizations with larger grains ($t \sim 25$ ns).

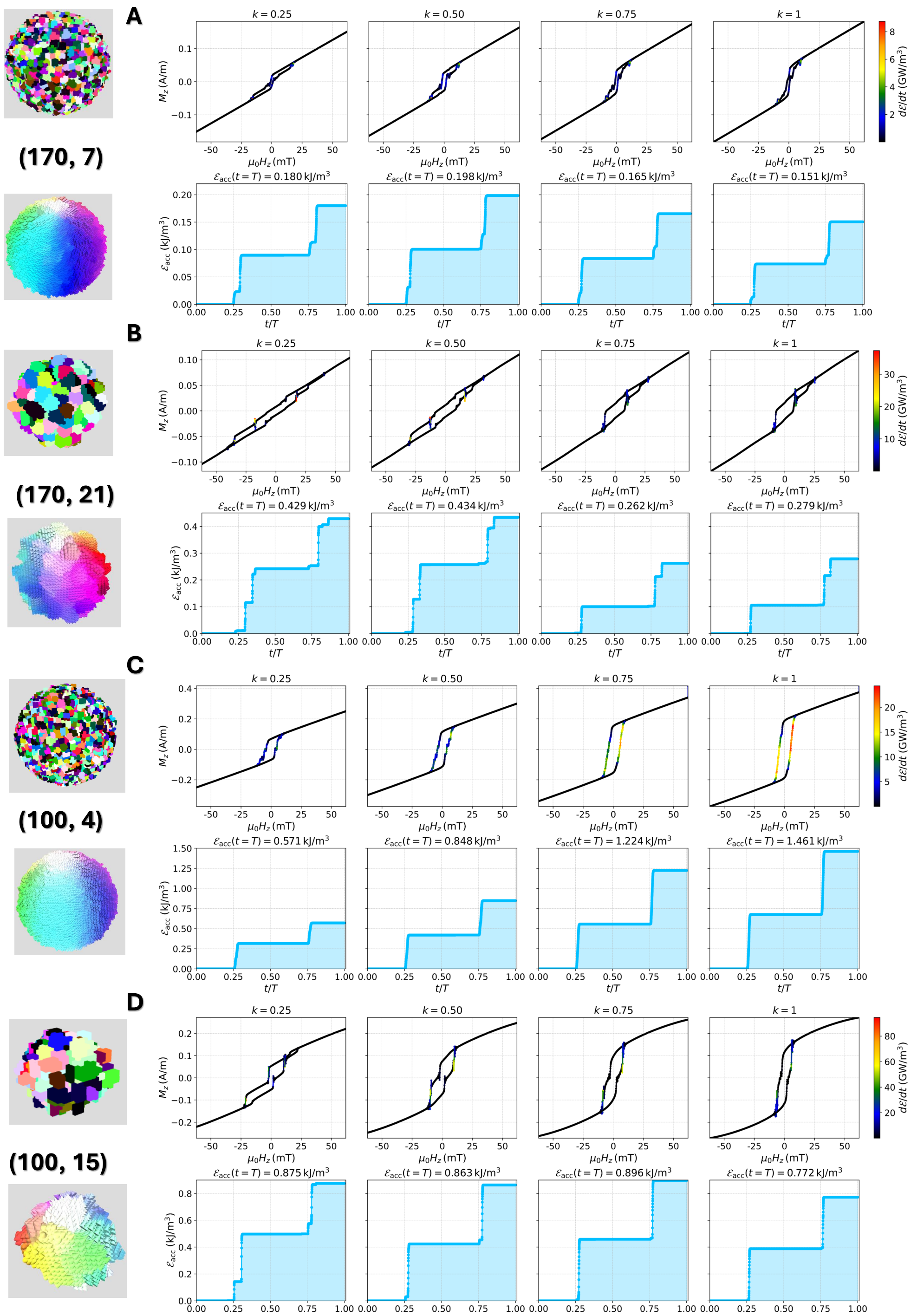

Figure S4: Magnetization as a function of applied field (top row) and $\Delta T(t)$ and $\mathcal{E}_{\mathrm{acc}}(t)$ (bottom row) over one AC-field period $T = 1/300$ ms for NFs with $k = 0.25, 0.5, 0.75$ and 1 and diameters of $d = 170$ nm and $g_s = 21$ nm (**A**) and 7 nm (**B**), and $d = 100$ nm and $g_s = 4$ nm (**C**) and 15 nm (**D**). The magnetization traces are colour-coded by the instantaneous heat generation, with red highlighting the maximum. Sketches represent grain anisotropy distribution (top), where each colour represents a $\vec{K_u}$ direction, and the vortex configuration (bottom), where each colour represents a direction of the magnetization.

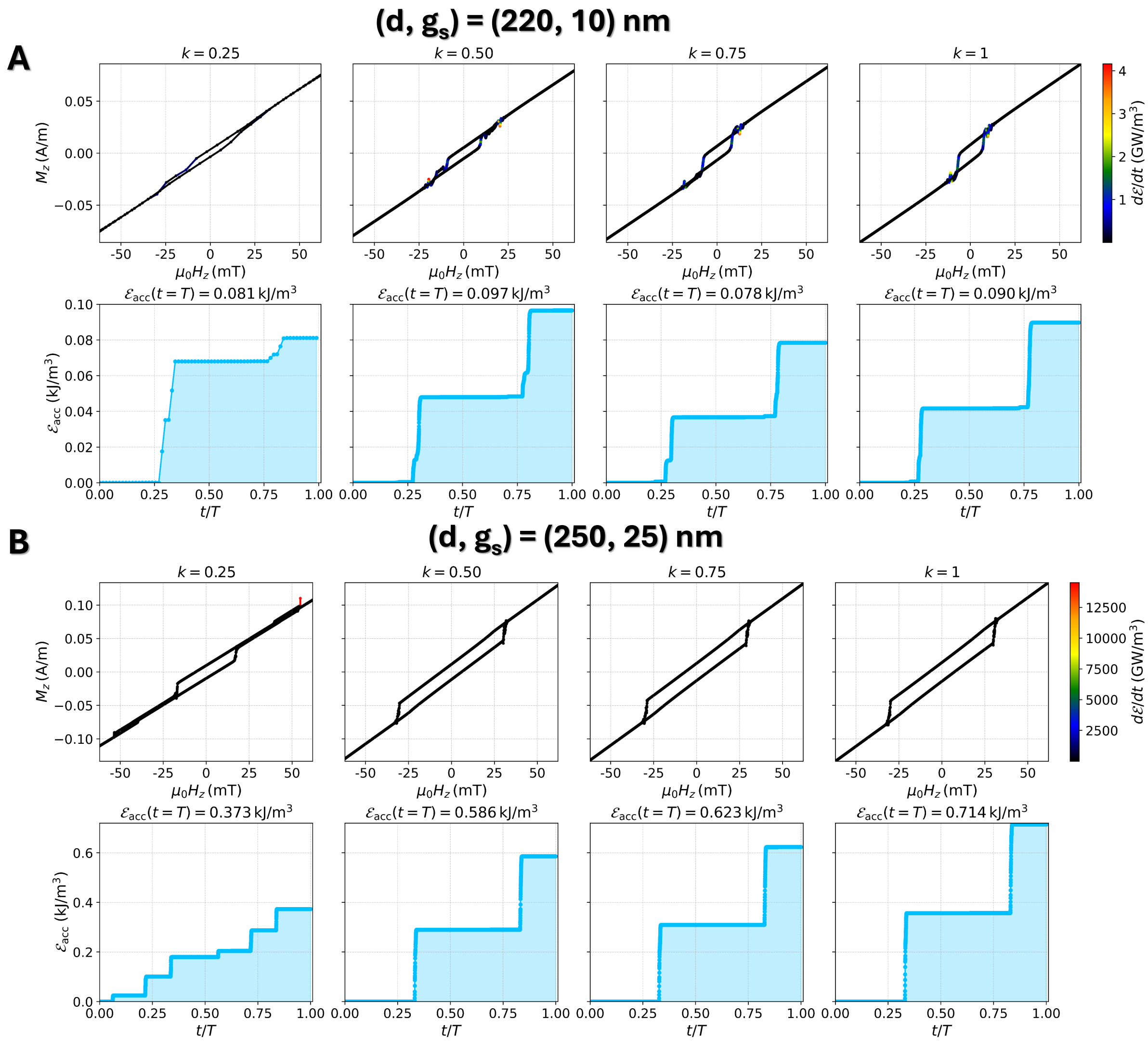

Figure S5: Magnetization as a function of applied field and corresponding released energy $\mathcal{E}(t)$ over one AC-field period $T = 1/300$ ms for NFs with diameters $d = 220$ nm and $g_s = 10$ nm (**A**), and $d = 250$ nm and $g_s = 25$ nm (**B**). The magnetization traces are colour-coded by the instantaneous heat generation, with red highlighting the points of maximum dissipation, coinciding with the jumps observed in the magnetization consequence of the reversal, *i.e.* moments of maximized torque. The step-like increases in $\mathcal{E}(t)$ coincide with these dissipation bursts. Inter-grain exchange is fixed to $k \times A$, with $k = 1.00$.

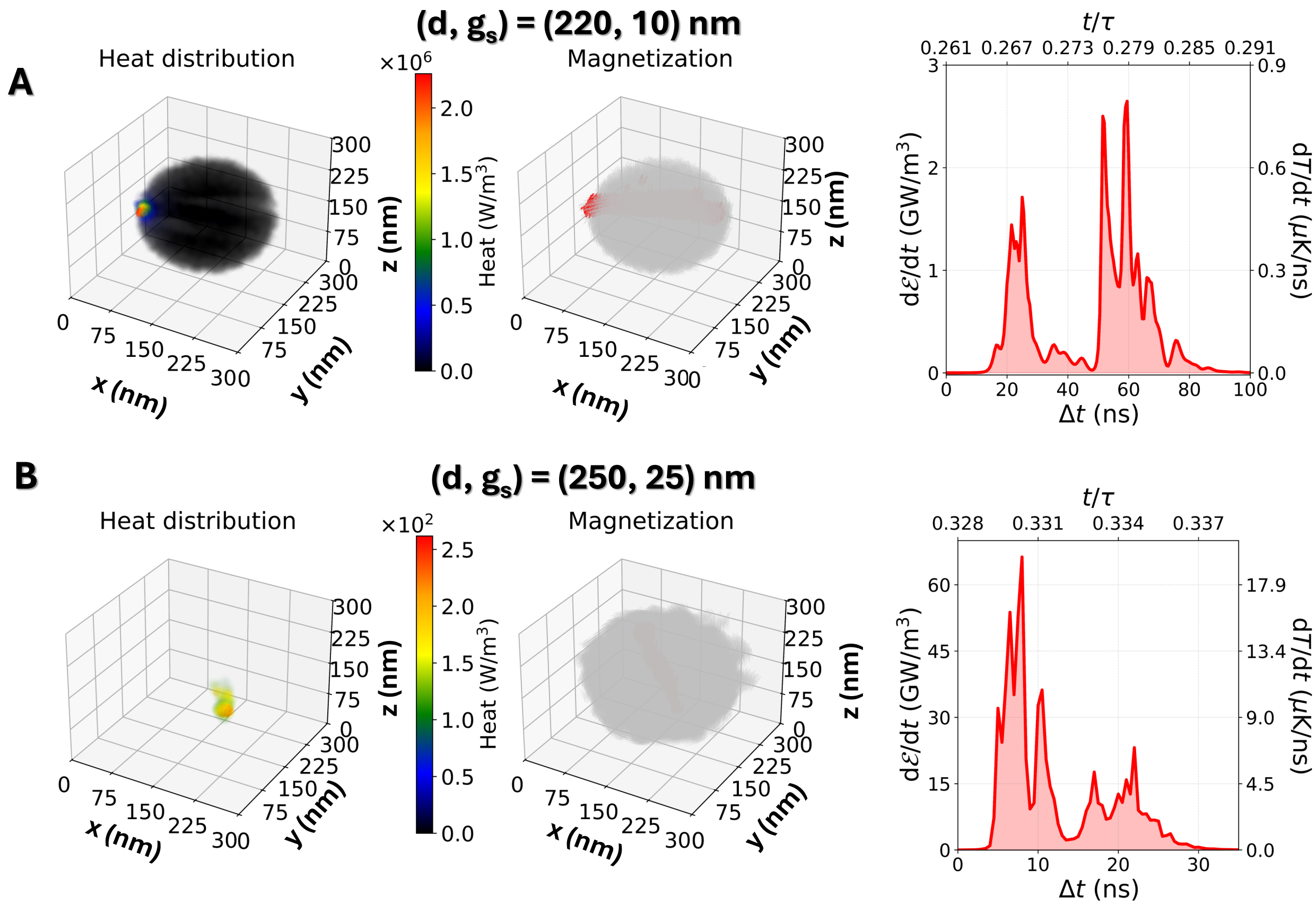

Figure S6: 3D heat and magnetization mappings, along with the instantaneous heat release $d\mathcal{E}/dt$ vs. Δt, for **(A)** $(d, g_s) = (220, 10)$ nm, and **(B)** $(d, g_s) = (250, 25)$ nm, respectively. Inter-grain exchange is fixed to $k \times A$, with $k = 1.00$. Top t/τ corresponds to $\tau = 3.33$ μs.

Instantaneous heat dissipation over a whole AC cycle

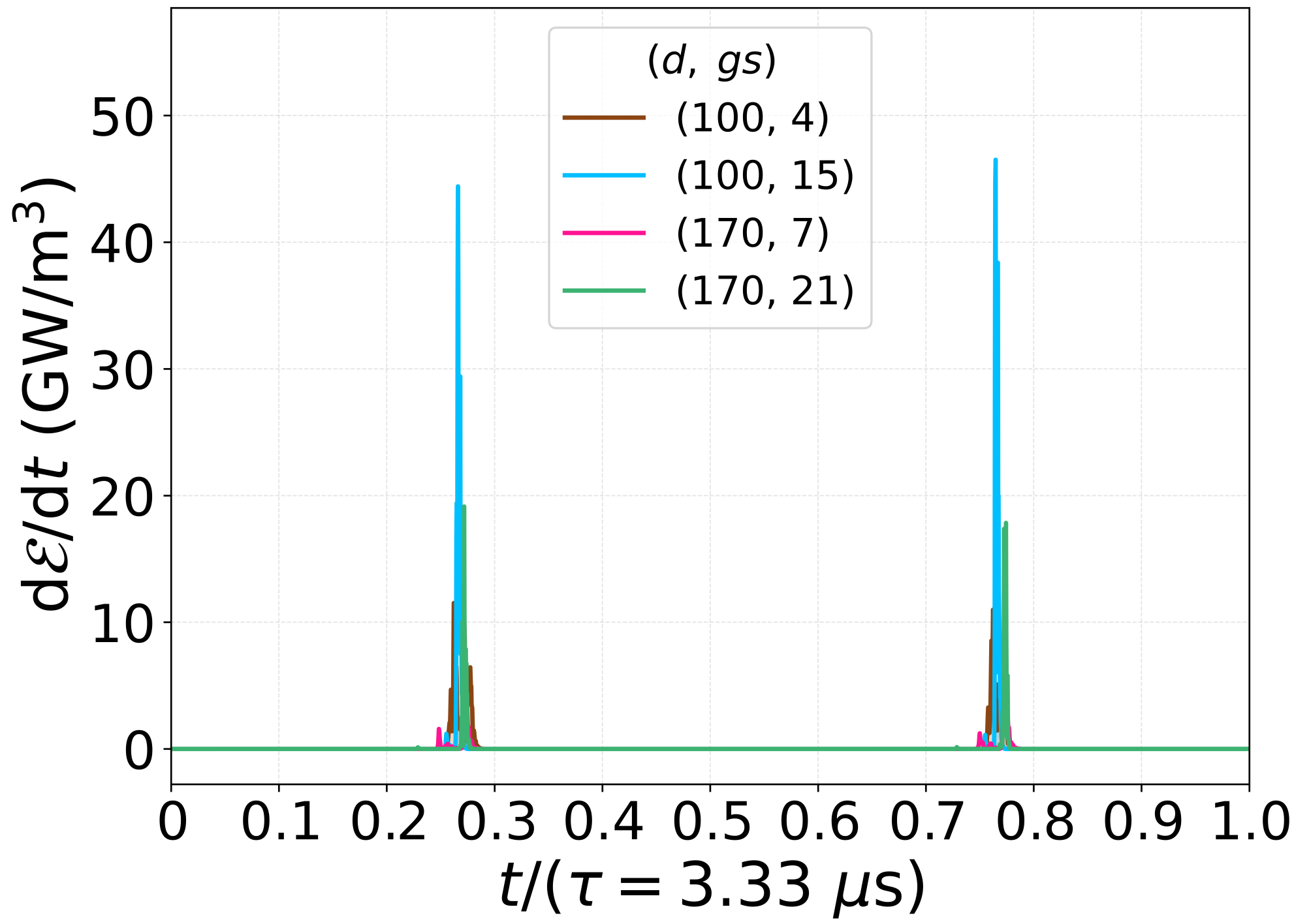

Figure S7: Instantaneous heat release $d\mathcal{E}/dt$ and corresponding dT/dt over a whole AC cycle ($\mu_0 H_Z = 62$ mT, $\tau = 1/f$ =3.33μs) for $(d, g_s) = (170, 7), (170, 21), (100, 4)$ and $(100, 15)$ nm at a fixed value of $k = 1$. The heat dissipation is concentrated over ∼100 ns time windows around $t = 0.25\tau$ and $t = 0.75\tau$.

Heat generation of a single domain spherical nanoparticle

In the main text, we have shown the influence of intra-particle disorder into the heat generation, underpinning how the heat is generated at specific intra-particle regions connected to pinning boundaries and over ns periods of time. Here we show how the heat is generated for an homogeneous particle (i.e., without intra-particle disorder). Figure S8 **(A)** includes the magnetization reversal as a function of the applied magnetic field and time for a single domain spherical nanoparticle. Regions marked as 1 and 3 correspond to the magnetization purely oriented along the z–axis (see **(B)**), whereas region 2 marks the area where the magnetization points perpendicular to the field direction. This region corresponds to the heat release, included in **(C)** as instantaneous heat release (gray line) and energy accumulated within the probed period. In **(D)**, the heating and magnetization mappings are shown for the moment of maximum heat release. Contrary to the vortex-like textures discussed in the work, the heat release of single-domain particles is homogeneous. Note the difference to Fig. 4 of the main text, where the heat release is purely concentrated along the vortex core direction, and extended over tens of nanoseconds, whereas in the single-domain case, the heat is released an order of magnitude faster ($t \sim 5$ ns).

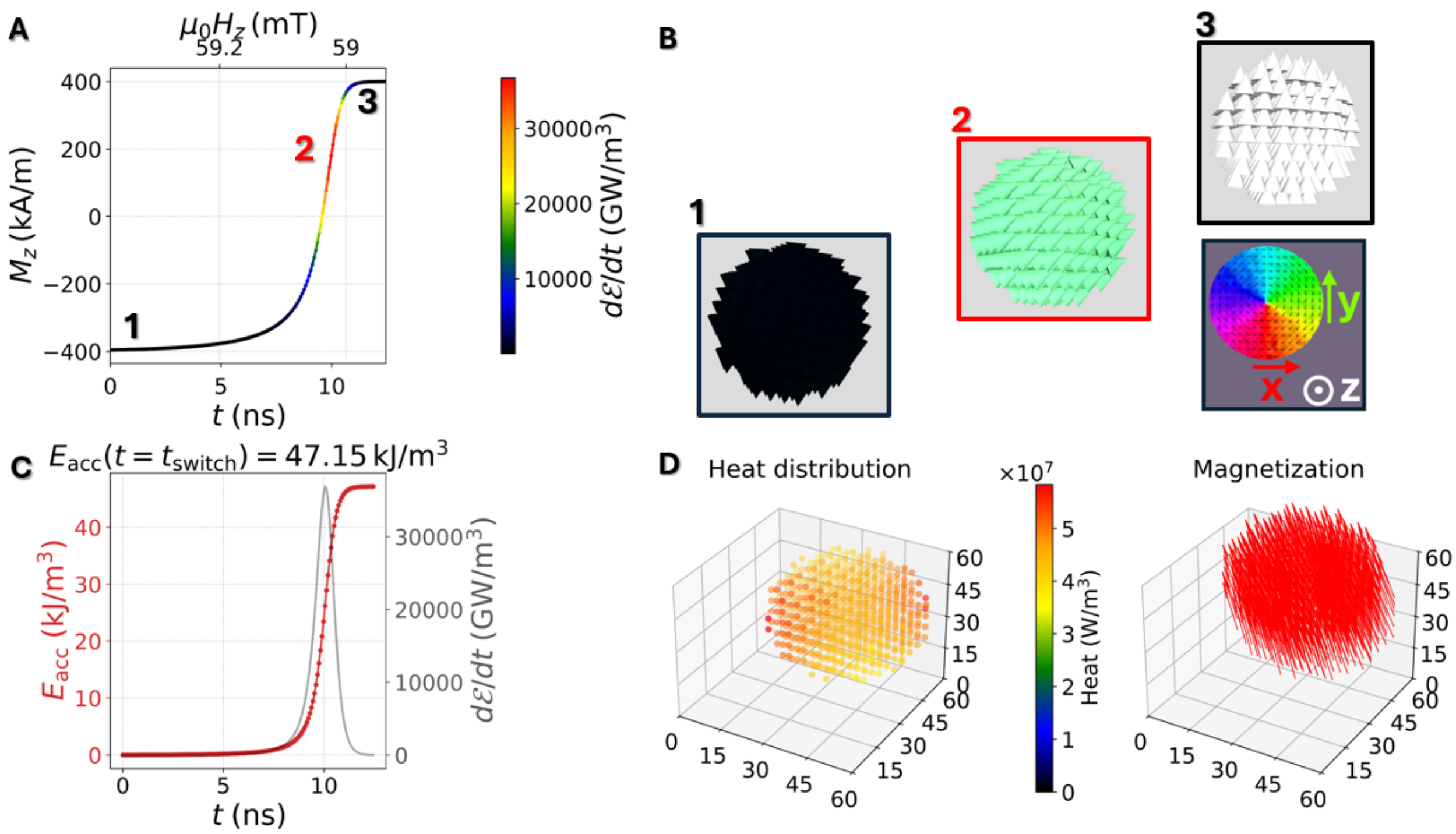

Figure S8: Single-domain 50 nm diameter nanosphere. **(A)** Magnetization as a function of applied field (top axis) and time (bottom axis). The magnetization traces are colour-coded by the instantaneous heat generation, with red highlighting the points of maximum dissipation. **(B)** Snapshots of three representative time steps of the magnetization reversal: 1 and 3 correspond to the magnetization along z-axis, 2 to the magnetization along the y direction. We have included the colour-coded wheel on the right side. **(C)** Instantaneous heat release (grey) and energy accumulated (red) for the reversal process, which happens within 5 nanoseconds. **(D)** temperature and magnetization mappings corresponding to the moment of maximum heat release. The heat is spread along the whole nanoparticle.

References

(1) Gavilán, H.; Sánchez, E. H.; Brollo, M. E. F.; Asín, L.; Moerner, K. K.; Frandsen, C.; Lázaro, F. J.; Serna, C. J.; Veintemillas-Verdaguer, S.; Morales, M. P.; Gutiérrez, L. Formation mechanism of maghemite nanoflowers synthesized by a polyol-mediated process. *ACS Omega* **2017**, *2*, 7172–7184.

(2) Hergt, R.; Dutz, S. Magnetic particle hyperthermia—biophysical limitations of a visionary tumour therapy. *Journal of Magnetism and Magnetic Materials* **2007**, *311*, 187–192.

(3) Hergt, R.; Dutz, S.; Zeisberger, M. Validity limits of the Néel relaxation model of magnetic nanoparticles for hyperthermia. *Nanotechnology* **2009**, *21*, 015706.

(4) Vansteenkiste, A.; Leliaert, J.; Dvornik, M.; Helsen, M.; Garcia-Sanchez, F.; Van Waeyenberge, B. The design and verification of Mumax3. *AIP Advances* **2014**, *4*, 107133.

(5) Leliaert, J.; Van de Wiele, B.; Vansteenkiste, A.; Laurson, L.; Durin, G.; Dupré, L.; Van Waeyenberge, B. Current-driven domain wall mobility in polycrystalline permalloy nanowires: A numerical study. *Journal of Applied Physics* **2014**, *115*, 233903.

(6) Gallo-Cordova, A.; Corrales-Pérez, B.; Cabrero, P.; Force, C.; Veintemillas-Verdaguer, S.; Ovejero, J. G.; P. Morales, M. Magnetic harvesting and degradation of microplastics using iron oxide nanoflowers prepared by a scaled-up procedure. *Chemical Engineering Journal* **2024**, *490*, 151725.

(7) Roca, A. G.; Marco, J. F.; Morales, M. P.; Serna, C. J. Effect of nature and particle size on properties of uniform magnetite and maghemite nanoparticles. *The Journal of Physical Chemistry C* **2007**, *111*, 18577–18584.

(8) Shokrollahi, H. A review of the magnetic properties, synthesis methods and applications of maghemite. *Journal of Magnetism and Magnetic Materials* **2017**, *426*, 74–81.

(9) Sinaga, E. P.; Adams, M. P.; Hasdeo, E. H.; Michels, A. Neutron scattering signature of the Dzyaloshinskii-Moriya interaction in nanoparticles. *Physical Review B* **2024**, *110*, 054404.

(10) Gross, B.; Philipp, S.; Josten, E.; Leliaert, J.; Wetterskog, E.; Bergström, L.; Poggio, M. Magnetic anisotropy of individual maghemite mesocrystals. *Physical Review B* **2021**, *103*, 014402.

(11) Borchers, J.; Krycka, K.; Bosch-Santos, B.; de Lima Correa, E.; Sharma, A.; Carlton, H.; Dang, Y.; Donahue, M.; Grüttner, C.; Ivkov, R.; Dennis, C. L. Magnetic Anisotropy Dominates over Physical and Magnetic Structure in Performance of Magnetic Nanoflowers. *Small Structures* **2025**, *6*, 2400410.

(12) Pisane, K.; Singh, S.; Seehra, M. Unusual enhancement of effective magnetic anisotropy with decreasing particle size in maghemite nanoparticles. *Applied Physics Letters* **2017**, *110*.

(13) Allia, P.; Tiberto, P. Dynamic effects of dipolar interactions on the magnetic behavior of magnetite nanoparticles. *Journal of Nanoparticle Research* **2011**, *13*, 7277–7293.

(14) Çam, N.; López-Vázquez, I.; Iglesias, Ò.; Serantes, D. Tuning field amplitude to minimise heat-loss variability in magnetic hyperthermia. *arXiv preprint arXiv:2601.20315* **2026**.

(15) Muñoz-Menendez, C.; Serantes, D.; Chubykalo-Fesenko, O.; Ruta, S.; Hovorka, O.; Nieves, P.; Livesey, K.; Baldomir, D.; Chantrell, R. Disentangling local heat contributions in interacting magnetic nanoparticles. *Physical Review B* **2020**, *102*, 214412.

(16) Leliaert, J.; Ortega-Julia, J.; Ortega, D. Individual particle heating of interacting magnetic nanoparticles at nonzero temperature. *Nanoscale* **2021**, *13*, 14734–14744.

(17) Ortega-Julia, J.; Ortega, D.; Leliaert, J. Estimating the heating of complex nanoparticle aggregates for magnetic hyperthermia. *Nanoscale* **2023**, *15*, 10342–10350.

(18) Guivar, J. A. R.; Martínez, A. I.; Anaya, A. O.; Valladares, L. D. L. S.; Félix, L. L.; Dominguez, A. B. Structural and magnetic properties of monophasic maghemite (γ-Fe_2O_3) nanocrystalline powder. *Advances in Nanoparticles* **2014**, *2014*.

(19) Collins, T. J. ImageJ for microscopy. *Biotechniques* **2007**, *43*, S25–S30.

(20) Cullity, B. D.; Smoluchowski, R. Elements of X-ray Diffraction. *Physics Today* **1957**, *10*, 50–50.

(21) Coey, J. M. D., *Magnetism and Magnetic Materials*, 1st ed.; Cambridge University Press: Cambridge, 2010.

(22) Di Fratta, G.; Serpico, C.; d'Aquino, M. A generalization of the fundamental theorem of Brown for fine ferromagnetic particles. *Physica B* **2012**, *407*, 1368–1371.

(23) Jefremovas, E. M.; Calus, L.; Leliaert, J. Coercivity-Size Map of Magnetic Nanoflowers: Spin Disorder Tunes the Vortex Reversal Mechanism and Tailors the Hyperthermia Sweet Spot. *Small Science* **2026**, *6*, e202500490.

(24) Coïsson, M.; Barrera, G.; Celegato, F.; Martino, L.; Kane, S. N.; Raghuvanshi, S.; Vinai, F.; Tiberto, P. Hysteresis losses and specific absorption rate measurements in magnetic nanoparticles for hyperthermia applications. *Biochimica et Biophysica Acta (BBA)-General Subjects* **2017**, *1861*, 1545–1558.

(25) Simeonidis, K.; Martinez-Boubeta, C.; Serantes, D.; Ruta, S.; Chubykalo-Fesenko, O.; Chantrell, R.; Oró-Solé, J.; Balcells, L.; Kamzin, A.; Nazipov, R.; Makridis, A.; Angelakeris, M. Controlling magnetization reversal and hyperthermia efficiency in core–shell iron–iron oxide magnetic nanoparticles by tuning the interphase coupling. *ACS applied nano materials* **2020**, *3*, 4465–4476.

(26) Myrovali, E.; Papadopoulos, K.; Charalampous, G.; Kesapidou, P.; Vourlias, G.; Kehagias, T.; Angelakeris, M.; Wiedwald, U. Toward the separation of different heating mechanisms in magnetic particle hyperthermia. *ACS Omega* **2023**, *8*, 12955–12967.

(27) Liu, X.; Zhang, Y.; Wang, Y.; Zhu, W.; Li, G.; Ma, X.; Zhang, Y.; Chen, S.; Tiwari, S.; Shi, K.; Zhang, S.; Ming Fan, H.; Xiang, Y.; Liang, X.-J. Comprehensive understanding of magnetic hyperthermia for improving antitumor therapeutic efficacy. *Theranostics* **2020**, *10*, 3793.
